# Impact of Serum Estradiol Levels Prior to Progesterone Administration in Artificially Prepared Frozen Embryo Transfer Cycles

**DOI:** 10.3389/fendo.2020.00255

**Published:** 2020-04-30

**Authors:** Shari Mackens, Samuel Santos-Ribeiro, Ellen Orinx, Neelke De Munck, Annalisa Racca, Caroline Roelens, Biljana Popovic-Todorovic, Michel De Vos, Herman Tournaye, Christophe Blockeel

**Affiliations:** ^1^Centre for Reproductive Medicine, Universitair Ziekenhuis Brussel (UZ Brussel), Brussels, Belgium; ^2^Research Group Reproduction and Immunology (REIM), Vrije Universiteit Brussel (VUB), Brussels, Belgium; ^3^IVI-RMA Lisboa, Lisbon, Portugal; ^4^IVI-RMA Abu Dhabi, Abu Dhabi, United Arab Emirates

**Keywords:** frozen embryo transfer, artificial cycle, estradiol, IVF/ICSI outcome, live birth rate

## Abstract

**Background:** The need for endocrine monitoring in artificial cycles for frozen embryo transfer (FET) remains unclear and, more specifically, the value of the late-proliferative phase serum estradiol (E2) levels is with conflicting evidence in current literature.

**Objective:** To investigate whether artificial FET cycles require endocrine monitoring for the serum E2 level prior to initiation of exogenous progesterone administration after an endometrial thickness of 6.5 mm has been reached.

**Design:** One thousand two hundred and twenty-two (*n* = 1,222) artificial FETs performed in a tertiary center between 2010 and 2015 were subdivided into 3 groups according to the following late-proliferative serum E2 level percentiles: ≤p10 (E2 ≤144 pg/ml; *n* = 124), p11–p90 (E2 from 145 to 438 pg/ml; *n* = 977) and >p90 (E2 >439 pg/ml; *n* = 121).

A mixed-effects multilevel multivariable regression analysis was performed to assess the potential effect of the late-proliferative E2 level on the live birth rate (LBR).

**Results:** The level of late-proliferative circulating E2 showed no significant difference in terms of LBR after FET. Specifically, the multivariable regression model demonstrated a LBR of 19.5% for the p11–p90 reference group, compared to 24.4% for the ≤p10 (*p* = 0.251) and 19.5% for the >p90 group (*p* = 0.989).

**Conclusion:** In this large retrospective dataset, no association was observed between late-proliferative phase serum E2 levels and LBR following FET in artificially prepared cycles. Although, caution is warranted due to the retrospective nature of the analysis and the potential for unmeasured confounding, we argue that monitoring of the late-proliferative serum E2 levels and using them to guide clinical decision-making (e.g., medication step-up, cycle prolongation or cancelation) may be of questionable value.

## Introduction

Although the use of FET has progressively increased, it is still unknown whether a specific endometrial preparation protocol should be favored over another (1, 2). Owing to their minimal need for clinic visits and a larger scheduling flexibility, artificial cycles for FET are widely used. Additional to the ultrasonographic endometrial thickness check prior to FET, the need for endocrine monitoring in artificial cycles remains unclear. Specifically, the value of proliferative phase serum hormone measurements remains a point of discussion.

Luteinizing hormone (LH) levels frequently rise in the proliferative phase of artificial cycles without pituitary suppression simulating that what occurs prior to a natural cycle ovulation. However, in this specific setting, the LH levels are no indicators for the phenomenon as a priori the estrogen (E2) supplementation suppresses follicular development (3, 4). Previous research has not shown an association of the mid-cycle LH level with the likelihood of pregnancy (5).

Late-proliferative progesterone (P) evaluation, on the other hand, is probably more reliable to detect escape ovulation; however, the actual need for measurement and its impact on final outcome is debatable as the occurrence of escape ovulation is rare, even in artificial cycles without gonadotropin releasing hormone (GnRH) agonist downregulation [1.9% according to (6)].

Proliferative phase serum E2 levels in artificial cycles for FET have also been investigated as they might have an impact on endometrial receptivity: estrogens stimulate endometrial proliferation and induce the expression of endometrial progesterone receptors which are necessary for the implantation of a transferred embryo (7). Several studies have described that mid-cycle serum E2 levels are not linked to ART outcome (8–11), while others reported that elevated mean and peak E2 levels throughout the proliferative phase may be negatively associated with ongoing pregnancy and live birth rates (LBRs) after artificial FET (12). An overview of the currently published literature with more detailed information on each individual study can be found in Table 1.

**Table 1:** Overview of the currently published literature.

**References**	**Study design,** **setting and size**	**Supplementation**	**Timing and assay**	**Outcome measures**	**Conclusion**
Remohi et al. (8)	Retrospective cohort Oocyte recipients 408 women 465cycles	Minimal 13 days of oral estradiol valerate in a step-up protocol with agonist co-treatment	Day of luteal phase induction Assay notspecified	Biochemical pregnancy rate Implantation rate Miscarriagerate	No impact
Banz et al. (9)	Retrospective cohort Conventional IVF/ICSI 325 women 325 cycles	14 days of transdermal 17β E2 patches without agonist co-treatment	Day before luteal phase induction Elecsys E2 II (Roche,Switzerland)	Clinical pregnancy rate Ongoing pregnancyrate	No impact
Niu et al. (10)	Retrospective cohort Conventional IVF/ICSI 136 women 176 cycles	14–20 days of oral estradiol valerate in a step-up protocol without agonist co-treatment	Day of luteal phase induction AXSYM assay (Abbott,USA)	Biochemical pregnancy rate Implantation rate Ongoing pregnancyrate	No impact
Bocca et al. (11)	Retrospective cohort Conventional IVF/ICSI 208 women 208 cycles	14 days of transdermal 17β E2 patches in a step-up protocol with in a later phase also vaginal administration without agonist co-treatment	Day after luteal phase induction Immulite 1000 (Siemens,PA)	Biochemical pregnancy rate Implantationrate	No impact
Fritz et al. (12)	Retrospective cohort Conventional IVF/ICSI 95 women 110 cycles	Minimal 14 days of transdermal or intramuscular E2 with or without extra vaginal supplementation, also with or without agonist co-treatment	Twice a week with E2 supplementation dosage adjustment to achieve a level of 200–500 pg/ml Immulite 2000 (Siemens,Germany)	Biochemical pregnancy rate Clinical pregnancy rate Ongoing pregnancyrate	Negative impact of elevated mean and peak levels

Based on these findings, we decided to further investigate this controversial topic by evaluating a large single-center 5-year retrospective cohort of identically prepared artificial FET cycles.

## Materials and Methods

### Ethical Approval

The study was approved by the Ethical Committee of the UZ Brussel (B.U.N. 143201835560) and performed in accordance with the endorsed guidelines.

### Study Design and Participating Patients/Cycles

For this retrospective analysis, all artificial FETs performed between January 2010 and December 2015 at the Center for Reproductive Medicine, Universitair Ziekenhuis Brussel, were extracted from the center's database. This specific time window was selected since, within this period, all embryos were cryopreserved using vitrification (before 2010 the center performed slow freezing), only one single hormone assay was applied to measure serum E2 levels and no changes were made to the center's artificial cycle preparation procedure. Following this primary extraction, cycles were excluded from the analysis if a GnRH agonist was added to the treatment, if the serum E2 level was not analyzed using the below-mentioned in-house assay, if the blood sampling performed was not 1 or 2 days before exogenously inducing the luteal phase, if the transferred embryo was generated using *in vitro* maturation, if estrogens were supplemented transdermally (instead of orally with estradiol valerate as described in detail below), if the patient was an oocyte donation recipient and/or if the FET cycle had been part of a clinical study investigating its most optimal timing (13, 14).

### Artificial FET Protocol

The same artificial protocol was applied to all cycles. Exogenous estrogens were administered orally under the form of estradiol valerate (Progynova®, Bayer-Schering Pharma AG, Berlin, Germany). Supplementation started on cycle day 2 after confirmation of basal serum hormonal values (for E2, P, and LH). Routinely, a dose of 2 mg was prescribed twice daily during the first 6 days, followed by 2 mg three times daily during the following 7 days. Cycle monitoring, with a first control visit planned on day 10–14 of endometrial preparation, included serum determinations of E2/P/LH and serial vaginal ultrasound scans. Serum P assessments were used to detect escape ovulation and the cycle was canceled when progesterone was 1.5 ng/ml or higher. If the endometrial thickness was below 6.5 mm on day 14 of supplementation, patients were asked to follow a step-up protocol consisting of (1) prolonging the duration of the estrogen administration but adhering to the same dose via the oral route, (2) increasing the dose but adhering the oral administration route, or (3) adding vaginal estrogen administration (also estradiol valerate at a dose of 2 mg three times daily) according to the physician's preference and experience. If following the step-up protocol endometrial thickness did not reach 6.5 mm, the cycle was canceled. Micronized progesterone (Utrogestan®, 200 mg three times daily; Besins Healthcare, UK) administered vaginally was used to induce the luteal phase. Once P supplementation was started, the estradiol valerate dose was decreased to 2 mg twice daily to mimic the natural cycle. Luteal phase support was continued until the time of the hCG pregnancy test and prolonged until 8 weeks of gestation whenever positive.

### Embryo Vitrification, Warming and Transfer

All embryos were vitrified on day 3 or day 5/6 of embryo culture using the same vitrification protocol, described in detail elsewhere (15).

Cleavage stage day 3 embryos were warmed the day before FET and transferred as a day 4 embryo on the fifth day of P supplementation. Blastocysts were warmed and transferred that same day on the seventh day of P supplementation.

In accordance with the Belgian legislation, one or maximum two embryos were transferred.

For day 3 vitrification, embryos were selected for cryopreservation if at least 6 blastomeres were present with ≤50% fragmentation. Morphological survival was assessed by counting the number of intact cells on the number of cells present at warming. Embryos with at least 50% of cells intact were considered surviving and further cultured. Further cleavage was assessed the next morning and defined as the percentage of embryos with at least division of 2 cells after overnight culture on the total number of transferred embryos (16). Blastocysts were vitrified on day 5 or 6 of embryo culture if they reached at least the full blastocyst stage with good quality inner cell mass and trophectoderm, i.e., at least type Bl3BB according to the Gardner scoring system (17). Blastocysts were warmed in the morning of the day of transfer and immediately evaluated for morphological survival. They were eligible for transfer if at least 50% of cells survived. The morphological quality of the blastocyst was scored at the moment of transfer. To account for embryo quality in the statistical analysis, we attributed a quality score from 1 to 4 to each transferred embryo (Supplementary Table 1).

### Serum E2 Measurement

Blood samples were drawn by venipuncture, allowed to coagulate at least 30 min followed by a centrifugation for 10 min at 1,800 g, after which serum was decanted, avoiding erythrocytes. Automated analysis was done by the hormone laboratory of the Universitair Ziekenhuis Brussel (Brussels, Belgium) with a validated laboratory immunoassay method (electrochemiluminescence, Cobas 6000®, generation II, Roche, Basel, Switzerland). The same assay was used during the entire study and regularly calibration was performed to minimize variation related to time and reagent batch renewal. Serum E2 levels were measured in all patients 1 or 2 days before the start of P supplementation for the induction of the luteal phase.

### Statistical Analysis

The artificial FET cycles were divided into three groups according to the *p* ≤ 10, p11–90, and *p* > 90 percentiles for late-proliferative serum E2 levels.

Comparisons between these groups for relevant patient background, fresh and frozen cycle characteristics were performed using mixed-effects regression in order to account for clustering of patients who performed more than one fresh cycle and, within these, more than one FET cycle, followed by pairwise comparisons whenever statistically significant.

LBR was the main outcome of the study. A mixed-effects multilevel multivariable regression analysis was performed to assess the potential effect of late-proliferative E2 adjusting for the following potential confounders: *maternal age at the time of embryo vitrification, body mass index, parity, indication for IVF/ICSI treatment, rank of the fresh cycle, number of usable embryos generated in the fresh cycle (i.e., suitable for fresh transfer or vitrification), biochemical pregnancy rate in the fresh cycle (in case of fresh transfer), rank of the FET cycle, presence of irregular cycles, cleavage or blastocyst stage transfer, single or double embryo transfer, embryo quality of the best frozen embryo transferred, endometrial thickness prior to FET, presence of follicular development despite artificial preparation and the for the FET cycle late-proliferative serum LH and P levels*. Adjusted odds ratios (aOR) and 95% confidence intervals (CI) were calculated.

A *p* < 0.05 was considered statistically significant and analyses were performed using STATA 13.1 (StataCorp. Stata Statistical Software: Release 13. College Station, Texas, USA) software.

## Results

A total of 854 patients were included in the analysis having performed 1,222 artificial autologous FET cycles. The *p* ≤ 10 group consisted of **124 cycles** with a minimal E2 level of 52 pg/ml and a maximal E2 level of 144 pg/ml (=p10). The p11–90 and *p* > 90 group included **977 cycles** with E2 values between 145 and 438 pg/ml and **121 cycles** with E2 values between 439 and 1,010 pg/ml, respectively.

### Characteristics of the Patients and IVF/ICSI Treatments

The baseline characteristics of the included patients and their fresh cycles are summarized in Table 2. Respectively, 98, 673, and 83 patients were included in the *p* ≤ 10, p11–90, and *p* > 90 groups. The groups did not differ significantly with regard to the majority of baseline characteristics, except for the indication for IVF/ICSI treatment and the rank of the fresh cycle. More specifically, unexplained infertility was more frequent in the *p* ≤ 10 group and the rank of the fresh cycle was significantly higher in the *p* > 90 group.

**Table 2:** Baseline patient and fresh cycle characteristics according to late-proliferative phase serum estradiol level percentiles of the FET cycle.

	***p* ≤ 10**	**p11–90**	***p* > 90**	***p*-value**
	***n*** **=** **98**	***n*** **=** **673**	***n*** **=** **83**	
Maternal age (years, mean ± SD)	31.0 ± 5.3	32.3 ± 4.6	34.0 ± 5.1	0.332
BMI (mean ± SD)	25.9 ± 5.0	25.1 ± 5.1	24.6 ± 5.0	0.473
Parity (mean ± SD)	0.4 ± 0.6	0.4 ± 0.6	0.3 ± 0.5	0.473
Presence of irregular cycles	43 (43.9%)	277 (41.3%)	35 (42.7%)	0.964
Indication for IVF/ICSI				
Male	21 (21.4%)	216 (32.1%)	24 (28.9%)	0.109
PCOS	12 (12.2%)	156 (23.2%)	18 (21.7%)	0.060
Endometriosis	5 (5.1%)	18 (2.7%)	2 (2.4%)	0.535
Tubal	8 (8.2%)	45 (6.7%)	6 (7.2%)	0.859
Ovulatory disorder	11 (11.2%)	49 (7.3%)	9 (10.8%)	0.260
PGT	27 (27.6%)	175 (26.0%)	29 (34.9%)	0.213
Unexplained	21 (21.4%)	86 (12.8%)	6 (7.2%)	0.017^*^^†^
Rank (mean ± SD)	1.8 ± 1.0	1.9 ± 1.4	2.3 ± 1.6	0.032^†^^‡^
Number of usable embryos (mean ± SD)	4.7 ± 2.9	4.9 ± 3.0	5.1 ± 2.4	0.681
Biochemical pregnancy rate (hCG positivity in case of immediate fresh transfer)	22 (33.3%)	165 (33.8%)	22 (31.4%)	0.925

*Analysis performed: mixed-effects modeling to account for clustering of patients who performed more than one cycle*.

*SD, standard deviation; BMI, body mass index; PCOS, polycystic ovary syndrome; PGT, preimplantation genetic testing: hCG, human chorionic gonadotrophin*.

*Freeze-all cycles excluded for the biochemical pregnancy rate calculation*.

*p < 0.05 for the following pairwise comparisons*.

* ≤ p10 vs. p11–p90;

† *≤ p10 vs. >p90; p11–p90 vs. >p90*.

The FET cycle characteristics can be found in Table 3. Of the analyzed parameters, two were significantly different between the groups: endometrial thickness was lower for *p* ≤ 10 when compared to p11–90 and follicular development despite estrogen supplementation was more prevalent for *p* > 90 when compared to p11–90.

**Table 3:** FET cycle characteristics according to late-proliferative phase serum estradiol level percentiles.

	***p* ≤ 10**	**p11–90**	***p* > 90**	***p*-value**
	***n*** **=** **124**	***n*** **=** **977**	***n*** **=** **121**	
Rank FET (mean ± SD)	1.4 ± 0.8	1.6 ± 1.0	1.6 ± 0.9	0.163
Blastocyst FET (as opposed to cleavage)	86 (69.4%)	642 (65.7%)	75 (62.0%)	0.681
Number of embryos warmed prior to FET (mean ± SD)	1.4 ± 0.6	1.5 ± 0.8	1.6 ± 0.7	0.110
DET (as opposed to SET)	40 (32.3%)	343 (35.1%)	52 (43.0%)	0.175
Best embryo transferred being of Q1 or Q2	98 (79.0%)	763 (78.1%)	88 (72.7%)	0.399
Total number of days of E2 supplementation prior to luteal phase induction (mean ± SD)	14.8 ± 3.8	14.0 ± 3.2	13.9 ± 3.5	0.053
Step-up protocol needed	32 (25.8%)	159 (16.3%)	21 (17.4%)	0.102
Endometrial thickness (mm, mean ± SD)	8.3 ± 1.7	8.8 ± 2.0	8.7 ± 2.1	0.041^*^
Late-proliferative serum LH (IU/l, mean ± SD)	14.0 ± 7.5	14.0 ± 9.2	13.4 ± 11.7	0.990
Late-proliferative serum P (ng/ml, mean ± SD)	0.6 ± 0.4	0.5 ± 0.4	0.5 ± 0.4	0.438
Follicular development despite E2 supplementation	3 (2.4%)	12 (1.2%)	9 (7.4%)	<0.001^‡^
Live birth rate	31 (25.0%)	207 (21.2%)	21 (17.4%)	0.373

*Analysis performed: multilevel mixed-effects modeling to account for clustering of patients who performed more than one fresh cycle and within these, more than one FET cycle*.

*SD, standard deviation; DET, double embryo transfer; SET, single embryo transfer; E2, estradiol; LH, luteinizing hormone; P, progesterone*.

*Q1 or Q2: see Supplementary Table 1 for definition*.

*p < 0.05 for the following pairwise comparisons:*.

* *≤ p10 vs. p11–p90; p11–p90 vs. >p90*.

### Unadjusted Analysis

**Figure 1:**
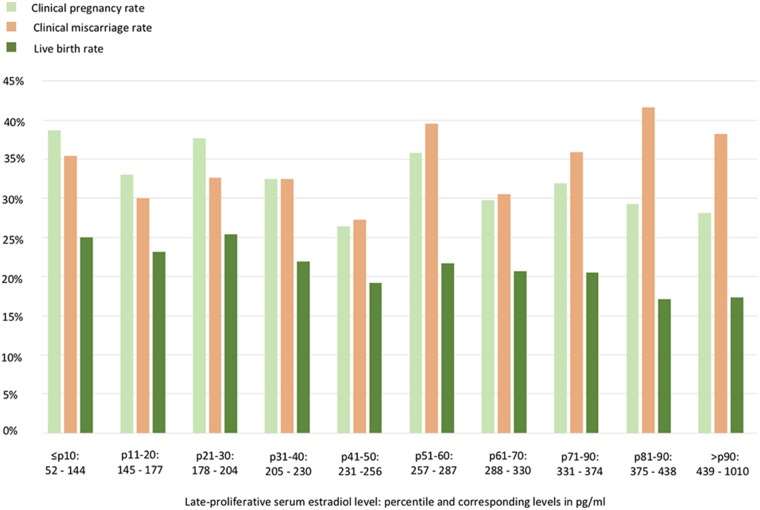
Artificial frozen embryo transfer cycle outcomes according to each 10th percentile of the late-proliferative serum estradiol level. No statistically significant relationship could be withheld.

LBRs were 25.0, 21.2, and 17.4% for the ≤ p10, p11–p90, and >p90 group, respectively (*p* = 0.373, Table 3). To assess further a possible relationship between the late-proliferative serum E2 level and the FET cycle outcome, the crude clinical pregnancy rate, clinical miscarriage rate and LBR are depicted in Figure 1 according to each 10th percentile. No statistically significant relationship was found.

### Adjusted Analysis

The aOR with the corresponding 95% CI and *p*-value are shown for each parameter included in the regression model in Table 4. The only significant *p*-value (0.001) was found at the level of the embryo quality score for the best embryo transferred (aOR 0.44, CI 0.27–0.72). The predicted LBR for the p11–p90 reference group was 19.5 %. For the ≤ p10 and the >p90 group, respectively, this was 24.4 and 19.5%, with no statistically significant difference.

**Table 4:** Mixed-effects multilevel multivariable regression model.

	**aOR**	**95% CI**	***p*-value**	**Predicted LBR**	
Late-proliferative serum E2 level					
≤ p10	1.35	0.81	2.25	0.251	24.4%
p11–p90	Reference				19.5%
>p90	1.00	0.55	1.82	0.989	19.5%
Maternal age	0.98	0.95	1.02	0.374	
BMI	0.99	0.96	1.03	0.708	
Parity	0.98	0.73	1.31	0.898	
Indication for IVF/ICSI					
Male	1.30	0.72	2.36	0.378	
PCO	0.79	0.38	1.66	0.534	
Endometriosis	1.74	0.66	4.62	0.266	
Tubal	1.27	0.57	2.83	0.553	
Ovulatory	0.87	0.34	2.25	0.781	
PGT	1.09	0.49	2.44	0.835	
Unexplained	1.81	0.86	3.81	0.119	
Presence of irregular cycles	0.79	0.56	1.12	0.180	
Rank fresh cycle	0.90	0.78	1.04	0.157	
Number of usable embryos	1.02	0.96	1.08	0.495	
Biochemical pregnancy during fresh embryo transfer					
No	Reference				
Yes	1.12	0.50	2.51	0.776	
No embryo transfer	0.68	0.36	1.29	0.235	
Rank FET cycle	1.00	0.80	1.25	0.994	
Blastocyst transfer (as opposed to cleavage)	1.08	0.73	1.59	0.715	
DET (as opposed to SET)	1.21	0.82	1.77	0.337	
Embryo quality score 1–2 for the best embryo transferred	0.44	0.27	0.72	0.001	
Endometrial thickness	1.08	1.00	1.17	0.052	
Late-proliferative serum LH	1.01	0.99	1.03	0.226	
Late-proliferative serum P	0.96	0.60	1.55	0.877	
Follicular development despite E2 supplementation	0.27	0.05	1.37	0.114	

*aOR, adjusted odd's ratio; CI, confidence interval; LBR, live birth rate; E2, estradiol; p, percentile; BMI, body mass index; PCO, polycystic ovaries; PGT, preimplantation genetic testing; FET, frozen embryo transfer; DET, double embryo transfer; SET, single embryo transfer; LH, luteinizing hormone; P, progesterone*.

*For the definition of embryo quality 1–2, see Supplementary Table 1*.

## Discussion

This single-center retrospective analysis did not detect a correlation between the serum E2 level prior to the induction of the luteal phase and the subsequent LBR in artificial FET cycles. To the best of our knowledge, this is the largest dataset addressing this particular question, consisting of more than double the size of the previously performed studies in terms of the included number of artificial FET cycles.

Its conclusion is in line with the majority of formerly published work (8–11), however, it is for the first time confirmed at the level of the LBR. Only in one report (12) a significant negative association was found between elevated proliferative phase serum E2 levels and ongoing pregnancy rate/LBR. This inconsistency between the studies could be the consequence of the difference in the artificial E2 supplementation protocol and/or the serum E2 evaluation performed (Table 1). Considerably, Fritz et al. included multiple artificial preparation protocols, adjusted the E2 supplementation dose in function of proliferative phase serum E2 level measurements and performed hormone analysis twice a week to calculate mean and peak values instead of focusing on one value at the time of luteal phase induction. High serum E2 values have indeed been associated with poorer outcome by others as well, however, they report mainly a higher risk for low birth weight/being small for gestational age in the setting of fresh embryo transfer following ovarian stimulation with clearly much higher E2 levels than the ones retrieved in artificial FET cycles (18, 19).

In this study, patients supplemented with E2 via the transdermal route were excluded, not because the administration route is thought to affect FET success rate (20), but as a different pharmacokinetic profile has been described (21). In our center, the above-mentioned artificial protocol using oral estradiol valerate is used routinely, while transdermal administration is applied mainly in second line or on specific patient's or treating physician's request. To avoid a possible bias, we chose to not include these cycles in the current analysis.

As the experimental results above show, few patients in an artificial FET cycle where the endometrium reaches 6.5 mm suffer from very low or high mid-cycle serum E2 levels. We opted to compare the ≤ p10 (with E2 levels ≤ 144 pg/ml) and the >p90 (with E2 levels >438 pg/ml) with the reference group p11–p90 in order to focus on the probably most biologically relevant very low and high values, while still preserving a sufficient sample size in each group. Indeed, a potential negative impact of even more extreme ranges of the spectrum is theoretically interesting and could not be excluded, however, the low prevalence of these events limits the importance for clinical practice. Besides this, another limitation of the study is the non-fixed time interval between the last estradiol dose intake and the harvesting of the blood sample to determine the circulating serum E2 level, which could have caused variation in the measurements.

When evaluating the background characteristics in this study, an interesting finding was the association between the late-proliferative phase serum E2 level percentiles and the endometrial thickness, showing a thinner endometrium in the ≤ p10 group compared to the p11–p90 group. This has been reported before for artificial preparation cycles in the oocyte recipient program (8), however, has not been confirmed in autologous FET cycles (9–11). Although a statistically significant association is seen in the current data, it has to be noted that an endometrial thickness below 6.5 mm was an indication for cycle cancellation and that such cycles are thus not included in this dataset. Furthermore, at the physiological level, the detected difference is anyway discrete and also not withheld when comparing the ≤ p10 with the >p90 group.

Another worth mentioning observation is the higher prevalence of follicular development in the >p90 group. This observation certainly makes physiological sense, nevertheless, it was only observed in 24 out of 1,222 cycles. The data showed once more that the natural cycle is adequately suppressed by early administered E2 supplementation and that, if serum P is used to detect escape ovulation, there seems to be no need for GnRH agonist co-treatment (6).

The only parameter with a statistically significant impact in the regression model was the embryo quality score for the best embryo transferred, underlining the importance of embryo quality for FET success. This association has been observed before in the context of slow-freezing protocols for cleavage stage (22) as well as for blastocyst FET (23, 24), although inter- and intra-observer agreement between embryologists in clinical practice has been reported to be suboptimal (25).

A final note belongs to the clinical miscarriage rate detected throughout this analysis (Figure 1). More specifically, after the confirmation of a clinical pregnancy, patients had an overall 34.4% risk of having a miscarriage. Although also euploid embryos can end in a miscarriage, it is important to mention that in this sample set no preimplantation genetic testing for aneuploidy (PGT-A) was routinely performed. The clinical miscarriage risk was also not found to be linked to the mid-cycle serum E2 level, however, deserves our attention as a high miscarriage rate following FET has been a point of debate in literature (26, 27). Furthermore, a possible stronger association with artificial cycle FET in comparison with natural cycle FET has been suspected (28, 29). A randomized controlled trial comparing the two preparation protocols is essential and is currently being conducted to provide conclusive answers (ClinicalTrials.gov identifier: NCT03976544).

Future research should definitely focus on the optimal luteal phase support in artificial FET cycles and the usefulness of serum P evaluation during this phase to optimize the success rate (30–32). Next to this, the obstetrical outcomes following artificial FET cycles, lying beyond the scope of this work, should be analyzed in depth. Recent studies have revealed a higher incidence of hypertensive disorders of pregnancy (HDP) in artificial FET cycles (33) and the underlying mechanism contributing to this increased obstetrical risk has not been entirely elucidated. Analysis of large series of artificial FET cycles should ultimately help unraveling the pathophysiology of HDP in artificial FET cycles.

In conclusion, no association between the mid-cycle serum E2 values and the subsequent LBR in artificial FET cycles was found. Therefore, the routine monitoring and use of these measurements to guide clinical decisions (e.g., medication step-up, cycle prolongation or cancellation) is of questionable value.

## Data Availability Statement

The datasets generated for this study are available on request to the corresponding author.

## Ethics Statement

The studies involving human participants were reviewed and approved by Ethical Committee of the UZ Brussel (B.U.N. 143201835560). Written informed consent for participation was not required for this study in accordance with the national legislation and the institutional requirements.

## Author Contributions

SM, SS-R, and CB designed the study and interpreted the data. SM and EO wrote the manuscript. SS-R performed the statistical analysis. EO was responsible for the creation of the database. ND, AR, CR, BP-T, MD, and HT contributed to the interpretation and editing of the manuscript. CB was responsible for the concept and supervised the study. All authors revised and approved the final version of the manuscript.

## Conflict of Interest

SS-R is currently employed by IVI-RMA, Lisbon, Portugal and ND by IVI-RMA, Abu Dhabi, UAE. Both authors were affiliated to the Center of Reproductive Medicine, Universitair Ziekenhuis Brussel (UZ Brussel), Brussels, Belgium at the time of the conducted research. The remaining authors declare that the research was conducted in the absence of any commercial or financial relationships that could be construed as a potential conflict of interest. The handling Editor declared a past co-authorship with one of the authors HT.
