# Supplementary material for: Impact of Serum Estradiol Levels Prior to Progesterone Administration in Artificially Prepared Frozen Embryo Transfer Cycles
Source: Front Endocrinol (Lausanne). 2020 Apr 30;11:255. doi: 10.3389/fendo.2020.00255 (PMC7204383; doi:10.3389/fendo.2020.00255)
Supplement: Supplementary file 1 [file Table_1.docx]

SUPPLEMENTARY TABLE 1. Embryo quality score prior to frozen embryo transfer

Cleavage stage embryos were warmed the evening before the day of transfer and left overnight in culture. Blastocyst stage embryos were warmed on the day of transfer.

Blastocyst developmental score (Bl1-6) and inner cell mass/trophectoderm score (A-D) according to the Gardner and Schoolcraft scoring system (Gardner and Schoolcraft *et al.,* 1999).

| EMBRYO QUALITY SCORE | Cleavage stage | Blastocyst stage^*^ |
| --- | --- | --- |
| 1 | Morula or Bl ≥1 | AA or BA |
| 2 | ≥5 cells with further cleavage | AB or BB |
| 3 | ≥5 cells with 1 more cell | AC, AD, BC, BD, CA, CB or Bl2 |
| 4 | ≥5 cells without further cleavage | CC, CD, DA, DB, DC, DD or Bl1 |

^*^Bl 3, 4, 5 or 6 unless mentioned otherwise.
